# Acute hepatitis B notification rates in Flanders, Belgium, 2009 to 2017

**DOI:** 10.2807/1560-7917.ES.2019.24.30.1900064

**Published:** 2019-07-25

**Authors:** Özgür Koc, Pierre Van Damme, Dana Busschots, Rob Bielen, Anmarie Forier, Frederik Nevens, Geert Robaeys

**Affiliations:** 1 Department of Gastroenterology and Hepatology, Ziekenhuis Oost-Limburg, Genk, Belgium; 2 Faculty of Medicine and Life Sciences, Hasselt University, Hasselt, Belgium; 3 Department of Medical Microbiology, School of Nutrition and Translational Research in Metabolism, Maastricht University Medical Centre, Maastricht, the Netherlands; 4 Vaccine and Infectious Disease Institute, Centre for the Evaluation of Vaccination, Antwerp University, Wilrijk, Belgium; 5 Department of Infectious Disease Control, Agency for Care and Health, Limburg, Belgium; 6 Department of Gastroenterology and Hepatology, University Hospitals KULeuven, Leuven, Belgium

**Keywords:** acute hepatitis B, hepatitis B vaccines, Belgium, prevention and control, blood-borne infections, sexually transmitted infections, hepatitis B virus, antenatal screening, men who have sex with men - MSM, public health policy, vaccines and immunisation, epidemiology

## Abstract

**Background:**

Belgium is a low-endemic country for hepatitis B. Universal hepatitis B vaccination in infants with catch-up in the age cohort of 10–13 year-olds began in 1999.

**Aims:**

Our objective was to evaluate the effect of prevention and control strategies on acute hepatitis B notification rates in Flanders (Belgium) from 2009 to 2017.

**Methods:**

This observational study collected demographic data and risk factors for acute hepatitis B from mandatory notifications to the Agency for Care and Health.

**Results:**

In Flanders, acute hepatitis B notification rates per 100,000 population decreased from 1.6 in 2009 to 0.7 in 2017. These rates declined in all age groups: 0–4-year-olds: 0.6 to 0.0, 5–14-year-olds: 0.2 to 0.0, 15–24-year-olds: 0.8 to 0.7, 25–34-year-olds: 3.4 to 1.1 and ≥ 35-year-olds: 1.59 to 0.7. There was also a downward trend in acute hepatitis B notification rates in native Belgians and first-generation migrants. Among 15–24-year-olds and 25–34-year-olds, a possible reversal of the decreasing trend was observed in 2016 and 2015, respectively. Among 548 acute hepatitis B cases, the main route of transmission was sexual activity (30.7%), and the pattern of transmission routes over time showed an increasing proportion of sexual transmission in men who have sex with men (MSM) after 2014. During the period from 2009 to 2017, five mother-to-child transmissions were reported.

**Conclusions:**

Prevention and control strategies were effective in reducing the acute hepatitis B notification rate. However, stronger prevention and control measures are needed in adult risk groups, particularly MSM.

## Introduction

Hepatitis B is caused by the hepatitis B virus (HBV), a member of the *Hepadnaviridae* family that infects liver cells, potentially causing liver cirrhosis and hepatocellular carcinoma [1]. Although most acute HBV infections are self-limiting, ca 0.1–0.5% of patients will develop fulminant hepatitis. Only 7–17% of 0–4-year-olds develop clinically apparent hepatitis after acute infection with HBV, compared with 29–35% for adults 30 years or older [2,3]. In addition to the relation between age and clinical hepatitis, there is a relation between age at infection and the likelihood of subsequently becoming a chronic hepatitis B patient. The risk of developing chronic HBV infection is estimated at 80–90% among infants infected perinatally if the mother is positive for hepatitis B e antigen, and this risk decreases with increasing age at infection [2,3].

HBV is widely prevalent and it is estimated that ca 2,000 million people have been exposed to the virus, with 257 million people living with chronic HBV infection worldwide [1]. In 2015, up to 887,000 deaths resulted from complications of hepatitis B, making it together with hepatitis C virus infection the 7th leading cause of mortality globally [1,4]. In Europe, the burden of hepatitis B differs by geographical region. Countries in eastern and southern Europe have a higher prevalence of chronic HBV infection than countries in the northern and western parts [5]. Belgium is classified as a country of low HBV endemicity, with ca 0.7% of the population positive for hepatitis B surface antigen (HBsAg) [6-8].

In Belgium, hepatitis B vaccine has been on the market since the 1982, yet universal free-of-charge vaccination only began in September 1999. Initially, infants received three consecutive doses from age 3 months, whereas adolescents received catch-up vaccination for the age cohort with the age range of 10–13 years. Since 2004, infants have been vaccinated with a hexavalent vaccine against diphtheria, tetanus, pertussis, hepatitis B, poliomyelitis and *Haemophilus influenza* type B at the ages of 8, 12 and 16 weeks and 15 months [9,10]. The catch-up vaccination in adolescents was concluded in 2012, as infants vaccinated in 1999 reached target age for adolescent vaccination in 2011 and 2012.

Reimbursement for hepatitis B vaccination in other birth cohorts is foreseen for certain patient groups (e.g. patients with haemophilia, pre-dialysis and dialysis patients, individuals with mental disabilities and first-degree relatives of a chronic hepatitis B patient), healthcare workers and employees who have an occupational risk for HBV infection. Hepatitis B vaccination is recommended but not reimbursed for the following groups: travellers to countries with intermediate or high HBV endemicity and specific HBV risk groups (e.g. men who have sex with men (MSM), sex workers, illicit drug users, patients with sexually transmitted infections (STI), individuals with multiple sex partners and patients with chronic liver disease).

In Belgium, there is endorsement for harm reduction programmes including peer support, opioid substitution therapy to reduce the frequency of injecting drugs and needle and syringe exchange programmes offering sterile injecting material for people who inject drugs [11]. Both healthcare workers and patients are encouraged to adhere to universal preventive methods and standard precautions to avoid exposure with HBV. There is also general advice to avoid unsafe sexual contacts through the use of condoms.

Strategies for screening have also been adopted. In Belgium, voluntary blood donors have been screened for HBV since 1972. Belgium has also had an antenatal HBV screening strategy since 2004 which is recommended during the first trimester of the pregnancy [12]. Children of HBsAg-positive mothers should receive the first hepatitis B vaccine together with a dose of 300 international units (IU) of hepatitis B immunoglobulins at birth [13].

The aim of the current study was to assess the impact of the above prevention and control strategies on acute hepatitis B notification rates in Flanders, Belgium. As the Belgian registration of hepatitis B did not make a distinction between acute or chronic hepatitis B until 2009, the main goal was to assess the trend in acute hepatitis B notifications from 2009 to 2017.

## Methods

### Study design

This observational study collected information on acute cases of viral hepatitis B in the period from 2009 to 2017 from the mandatory notifications to the Agency for Care and Health (Flanders). In Flanders, hepatitis B has been notifiable since 1971, and information collected by the Agency for Care and Health includes diagnosis, basic demographic data (e.g. age, sex and country of birth), clinical features, serological test results and risk factors for infection on the date of diagnosis. Before 2009, a differentiation between acute or chronic hepatitis B was not possible. Since then, only acute hepatitis B has been reported by mandatory notifications from both laboratories and physicians to the Agency for Care and Health (Flanders).

### Outcome measures

The primary study endpoint was the assessment of the overall trend in acute hepatitis B notification rates in Flanders from 2009 to 2017. Secondary and exploratory endpoints were the acute HBV notification rates by age, sex and country of birth. We also assessed the main routes of transmission among our study population of acute hepatitis B cases.

#### Case definition

In our criteria for differentiating acute and chronic hepatitis B, patients with the appropriate symptoms and signs (e.g. jaundice, elevated serum aminotransferase levels, abdominal pain, loss of appetite, nausea, vomiting, fatigue or fever) and laboratory confirmation were identified as definite acute hepatitis B cases [14]. Laboratory confirmation included (i) a positive test for HBsAg and anti-HBc IgM, (ii) detection of HBsAg and negative HBsAg in the 6 months before presentation which are routinely reported if available or (iii) detection of HBV DNA and negative for HBV DNA in the 6 months before presentation which are routinely reported if available [14]. In line with the European Union case definitions, unknown cases were identified as newly diagnosed HBsAg-positive individuals with the appropriate symptoms and signs but without IgM determination or previous markers for HBV infection [14].

#### Notification rates and vaccination coverage

According to the population data from Statistics Belgium (StatBel), the total population in Flanders increased from 6,208,877 in 2009 to 6,516,011 in 2017 [15]. Acute hepatitis B notification rates from 2009 to 2017 were calculated per 100,000 population and were based primarily on the total number of acute hepatitis B notifications, i.e. the sum of definite and unknown acute HBV cases. Age groups were categorised as 0–4, 5–14, 15–24, 25–34 and ≥ 35 years on mid-decade to mid-decade. The ≥ 35-year-olds were individuals not covered by the universal hepatitis B vaccination with catch-up in children aged 10–13 year in Flanders. Based on the country of birth, infected persons were identified as Belgian or as first-generation migrants (FGM), i.e. born outside Belgium. FGM were grouped according to the six World Health Organization (WHO) regions: Africa, the Americas, the Eastern Mediterranean, Europe, South East Asia and the Western Pacific [16].

In this study, vaccination coverage data for infants and adolescents in Flanders were obtained from an extended programme of immunisation-based surveys [17-21]. For calculating the influence of prevention and control strategies on the age distribution of acute hepatitis B patients over time, we stratified notification rates into two time periods, 2009–12 and 2013–17. The cut-off was chosen to balance the time covered, and 2012 was the end of the catch-up period for adolescents.

### Statistical analysis

Binary data were analysed using chi-squared test or Fisher’s exact test. Comparison of two continuous variables was done with the independent t-test or Mann–Whitney U-test, depending on the presence of normality and homogeneity. Results are presented either as frequencies (%) or as means (standard error of mean (SEM)). The level of statistical significance was set at p < 0.05.

### Ethical statement

Following Belgian regulation, ethical approval was waived because of the non-interventional character of our study.

## Results

### Overall trend

**Figure 1:**
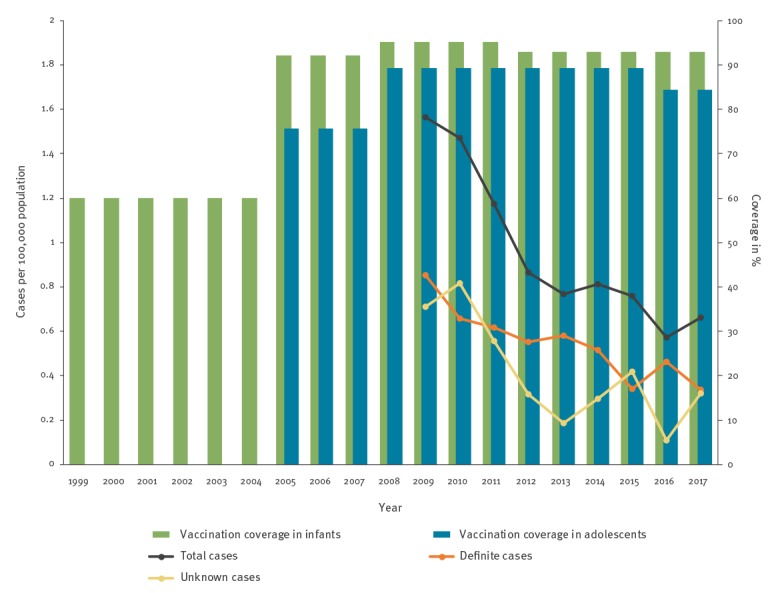
Acute hepatitis B notification rates, 2009–2017 (n = 548) and coverage with a complete hepatitis B vaccine schedule, 1999–2017, Flanders

Acute hepatitis B notification rates decreased by 2017 to 42%, 40% and 55% of the 2009 rate for, respectively, the total number of cases, definite cases and unknown cases (Figure 1). The hepatitis B incidence for the total number of cases in Flanders declined from 1.56 per 100,000 population in 2009 to 0.77 per 100,000 population in 2013 and 0.66 per 100,000 population in 2017. For definite and unknown cases, the respective incidences were 0.85 and 0.71 per 100,000 population in 2009, 0.58 and 0.19 per 100,000 population in 2013, and, 0.34 and 0.32 per 100,000 population in 2017. Reported hepatitis B vaccination coverage among infants between 1999 and 2017 is also presented in Figure 1. In infants, coverage with three doses of hepatitis B vaccine exceeded 90% from the 2005 survey onwards in Flanders. In adolescents, the HBV vaccination coverage was below 90% up to 2017 (Figure 1).

Definite cases were defined as patients with the appropriate symptoms/signs and laboratory confirmation, i.e. a positive test for HBsAg and anti-HBc IgM, detection of HBsAg and negative markers in the 6 months before presentation or detection of HBV DNA and negative HBV markers in the 6 months before presentation. Unknown cases were defined as HBsAg-positive patients with the appropriate symptoms/signs without IgM determination or previous markers for HBV infection. Total cases are the sum of definite cases and unknown cases.

Estimates on vaccination coverage in infants for the period 2000–04 were based on survey results from 1999. Survey results from 2005, 2008, 2012 and 2016 were used to determine vaccination coverage in infants and adolescents for the periods 2006–07, 2009–11, 2013–15 and 2017.

### Age and sex

**Figure 2:**
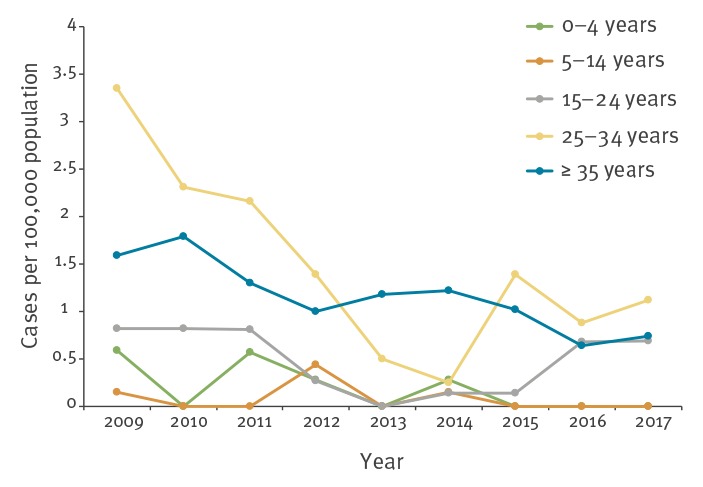
Acute hepatitis B notification rate per 100,000 population by age group, Flanders, 2009–2017 (n = 548)

Notification rates by age groups from 2009 to 2017 are presented in Figure 2. The rates in 2009 for age groups 0–4, 5–14, 15–24, 25–34 and ≥ 35 years were 0.59, 0.15, 0.82, 3.35 and 1.59 per 100,000, respectively. In the period 2009 to 2017, the data clearly show that the trend in hepatitis B notification rates was decreasing for all age groups. The notification rates in 2017 for the 0–4, 5–14, 15–24, 25–34 and ≥ 35 year-olds were 0.00, 0.00, 0.68, 1.12 and 0.74 per 100,000, respectively. There was an increase in incidence among those aged 15–24 years and 25–34 years in 2016 and 2015, respectively.

**Figure 3:**
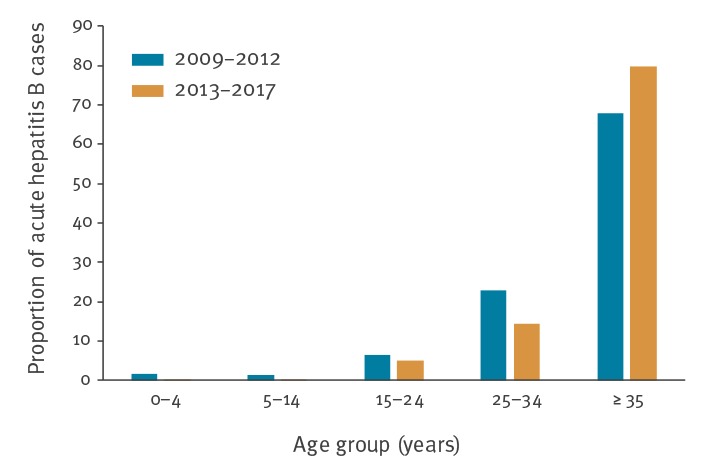
Proportion of acute hepatitis B cases by age group in 2009–2012 and 2013–2017, Flanders (n = 548)

Figure 3 shows the age distribution of acute hepatitis B notifications in the periods 2009 to 2012 and 2013 to 2017. There was a decrease in the proportion of 15–24 year-olds from 6.4% between 2009 and 2012 to 5.2% between 2013 and 2017 (p = 0.597). For the 25–34 year-olds, this was 22.9% and 14.3%, respectively (p = 0.015). Moreover, the mean age of the acute hepatitis B cases in the period 2009 to 2012 was significantly lower than in the period 2013 to 2017 (43.0 ± 0.99 years vs 46.6 ± 0.96 years; p = 0.013).

**Figure 4:**
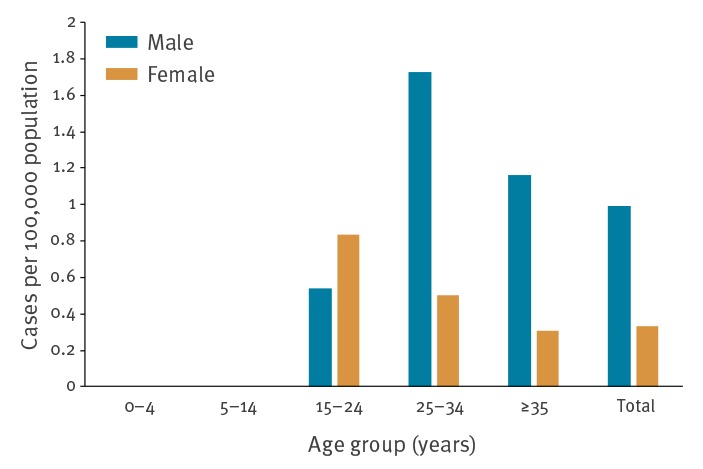
Acute hepatitis B notification rate per 100,000 population by age group and sex, Flanders, 2017 (n = 43)

Notification rates for male and female cases in 2017 were 0.99 and 0.33 per 100,000, respectively. The rates in adolescents and young adults were slightly higher among women, whereas men had higher rates than women in the age groups 25 years and older. Acute hepatitis B notification rates in 2017 were highest among 25–34-year-old men (Figure 4). This age group had the highest rate difference between male and female cases (1.73 and 0.50 per 100,000 in 2017, respectively).

The notification rates for male and female cases were, respectively, 2.14 and 0.86 per 100,000 in 2009 and 1.05 and 0.50 per 100,000 in 2013. In the age group 25 years and older, the notification rates in men remained higher than in women throughout the study period (data not shown).

### Country of birth

Country of birth data were available for 455 of 548 (83.0%) acute hepatitis B cases. Among those 455 individuals, 360 (79.1%) were born in Belgium. Among FGM, 51 (53.7%) originated from the WHO European Region (13 from Turkey, eight from Romania and 30 from 16 other countries), 17 (17.9%) from the WHO African Region (three from Ghana and 14 from 10 other countries), 15 (15.8%) from the WHO Eastern Mediterranean Region (five from Morocco, four from Afghanistan and six from six other countries), six (6.3%) from the WHO South East Asian Region (three from Indonesia and three from two other countries) and six (6.3%) from the WHO Western Pacific Region (three from China and three from two other countries).

**Figure 5:**
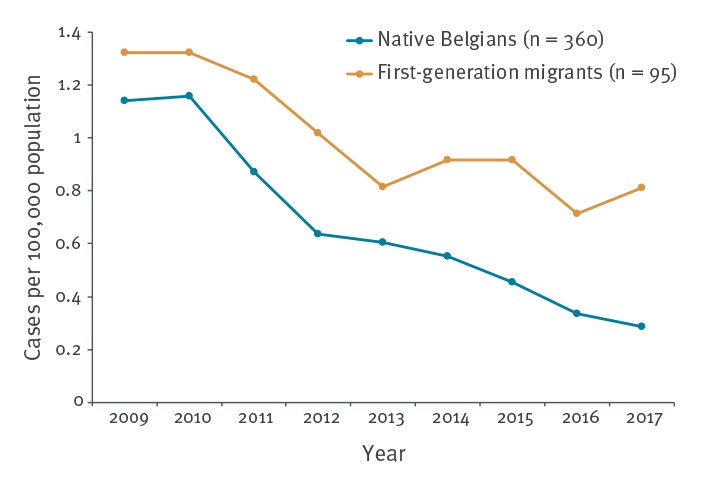
Acute hepatitis B notification rate per 100,000 population by country of birth, Flanders, 2009–2017 (n = 455)

The age structure between native Belgians and FGM was different, namely 16 (4.4%) vs 16 (16.8%) 0–24-year-olds (p < 0.001), 61 (16.9%) vs 31 (32.6%) 25–34-year-olds (p < 0.001) and 283 (78.6%) vs 48 (50.5%) ≥ 35-year-olds (p < 0.001). Figure 5 illustrates a downward trend over time in acute hepatitis B notification rates in native Belgians as well as in FGM. The incidence of acute hepatitis B was higher among FGM and the gap between the two groups increased after 2013.

### Routes of transmission

**Figure 6:**
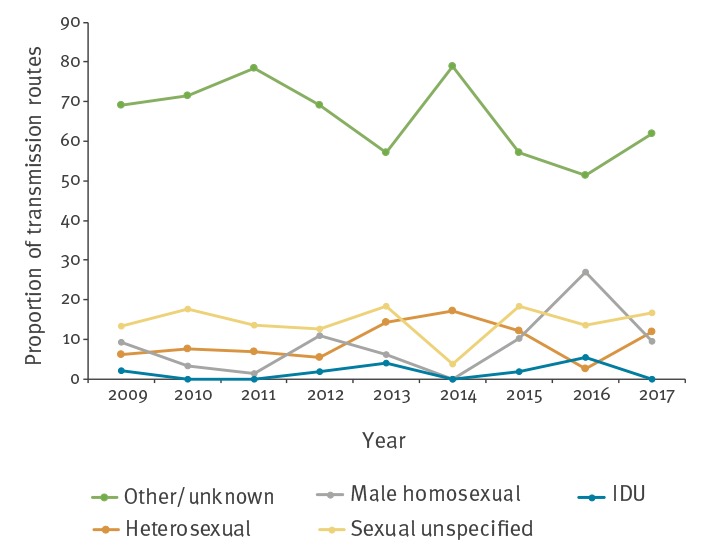
Pattern of transmission routes for acute hepatitis B notifications, Flanders, 2009–2017 (n = 548)

Information on routes of transmission was available in 213 (38.9%) of the 548 acute hepatitis B notifications. Over the time period from 2009 to 2017, the main route of transmission for acute hepatitis B was sexual activity in 168 cases (78.9%), of whom the majority did not specify sexual interest (78/168, 46.4%). Forty-nine (29.2%) of 168 individuals reported heterosexual activity and 41 (24.4%) reported male homosexual activity. Intravenous drug use accounted for eight (3.8) of the 213 cases (Table). The Table also illustrates the routes of transmission by country of birth. All five (2.3%) reported mother-to-child transmissions occurred in Belgium. Transmission through sexual activity was more common in men than in women (131/162 (80.9%) vs 37/51 (72.5%), p = 0.300), and 41 (25.3%) were MSM. The routes of transmission for acute hepatitis B cases over time are illustrated in Figure 6. In particular, an increasing trend of male homosexual transmission was apparent from 2014 onwards.

**Table:** Routes of transmission for acute hepatitis B cases, Flanders, 2009–2017 (n = 213)

Routes of transmission	Total (n = 213)		Native Belgian (n = 170)		FGM (n = 24)		p value
	n	%	n	%	n	%	
Intravenous drug use	8	3.8	5	2.9	3	12.5	0.067
Sexual	168	78.9	135	79.4	16	66.7	0.097
- Heterosexual	49	23.0	42	24.7	5	20.8	0.618
- Male homosexual	41	19.2	35	20.6	2	8.3	0.176
- Sexual, unspecified	78	36.6	58	34.1	9	37.5	0.838
Mother-to-child	5	2.3	3	1.8	0	0.0	1.000
Invasive healthcare procedure/dental	7	3.2	5	2.9	2	8.3	0.220
Blood transfusion/transplantation	3	1.4	3	1.8	0	0.0	1.000
Needle injury or other occupational exposure	3	1.4	3	1.8	0	0.0	1.000
Tattooing/body piercing	3	1.4	2	1.2	1	4.2	0.337
Other	16	7.5	14	8.2	2	8.3	1.000

FGM: first-generation migrants.

Information on routes of transmission was not available in 335 of 548 (61.1%) overall, in 190 of 360 (52.8%) native Belgians and 71 of 95 (74.7%) FGM.

IDU: intravenous drug use.

Compared with native Belgians, FGM were more frequently infected abroad (21/285 with known information on place of transmission and country of birth (7.4%) vs 20/61 (32.8%); p < 0.001). Among the 20 FGM infected abroad, 16 were infected while travelling in their country of birth. Among the individuals who acquired acute HBV infection abroad (44/398 with known information on place of transmission; 11.1%), 36 acquired it in an intermediate or high HBV-endemic country: 15 in the WHO European Region, 11 in the WHO South East Asian Region, four in the WHO African Region, three in the WHO Western Pacific Region, two in the WHO Eastern Mediterranean Region and one in the WHO Region of the Americas.

It was reported that 40 individuals acquired HBV infection by heterosexual transmission in Belgium and six abroad. For male homosexual and unspecified sexual transmission, the numbers were 32 vs four and 59 vs six, respectively.

## Discussion

The acute hepatitis B notification rates in Flanders Belgium decreased from 2009 to 2017 in all age groups (0–4 years, 5–14 years, 15–24 years, 25–34 years and ≥ 35 years). Since universal infant vaccination started in 1999, notification rates for 5–14-year-olds from 2013 onwards represent age groups that received universal infant vaccination, which may explain the decline in acute hepatitis B notification rates after 2013 in this age group. Moreover, the downward trend in 15–24- and 25–34-year-olds indicates the birth cohorts 1987 to 1998 who were covered by universal catch-up vaccination in this low-prevalence country where sexual activity is recognised as the most important exposure for HBV infection [22].

The effects of a universal HBV vaccination programme in infants with catch-up in adolescents were also evaluated on serum samples collected in 2006 and 2007 from children aged 1–19 years by Theeten et al. [10]. They demonstrated that the prevalence of HBV infection remained low in Belgium and that overall high levels of vaccine-induced immunity were achieved in infants as well as in adolescents.

Although the hepatitis B vaccination coverage in infants and toddlers for the three doses increased above 90% after 2004, the continuing downward trend in acute hepatitis B notification rates in ≥ 35-year-olds from 2009 to 2017 cannot be explained by universal infant and toddler vaccination with catch-up in adolescents. For this, an explanation should be explored in the vaccination and health behaviour of certain patient groups (e.g. dialysis patients), healthcare workers, those with occupational risk of HBV infection, travellers to intermediate or high HBV-endemic countries, patients with STI and sex workers in Belgium. However, there is no information on the uptake of vaccination in adults aside from Belgian studies in sex workers, healthcare workers, travellers to intermediate or high HBV-endemic countries and pregnant women [23-28]. In order to eliminate hepatitis B as a public health threat by 2030 as recommended by WHO, recent studies are warranted to evaluate hepatitis B prevention and control measures, particularly in adult risk groups such as MSM and patients with STI [29].

Moreover, relatively higher notification rates of acute hepatitis B were observed in adults than in children, emphasising the importance of raising the awareness of healthcare workers, policymakers and the general public about stronger prevention and control measures targeting the adult group. These numbers could be ascribed to (i) vaccination uptake and/or (ii) sexual transmission being the most important infection route for hepatitis B in a low-prevalence country and/or (iii) the relation between age and clinical hepatitis [3,10,22].

By 2018, 191 of 194 (98%) WHO member states had introduced universal infant or childhood hepatitis B vaccination. This is a substantial increase compared with the year 2000 where only 129 (66%) countries had included hepatitis B vaccination in their national universal immunisation system [30]. In this context, we found that acute hepatitis B notification rates declined also in FGM, not only in native Belgians. However, the reduction in acute hepatitis B cases among FGM was smaller than among native Belgians; therefore, the number of FGM with acute hepatitis B could have limited the overall decreasing trend of acute hepatitis B cases found in the current study. Our findings are in line with a previous study indicating that the estimated number of vaccinated people is lower for FGM than for native Belgians [6]. In view of the increase in hepatitis B incidence among FGM in 2017, it should be noted that this trend could possibly continue after 2017. There is currently no screening programme targeting this group, although vaccination is recommended but not reimbursed for immigrants travelling back to their endemic country of birth. New infections among adult FGM and their relatives could be prevented.

After 2013, the decline in hepatitis B incidence was less pronounced. This could possibly be related to the bimodal pattern of immunisation coverage with the highest proportion of vaccinated individuals in the birth cohorts targeted by the vaccination programme (1999–2002 and 1987–1989) with a clear dip in between [10]. Moreover, there was a possible reversal of the decreasing trend for 15–24-year-olds and 25–34-year-olds in 2016 and 2015, respectively. This could in part be ascribed to a rise in the number of cases among MSM. Notification rates by sex indicated that rates were higher in men with the highest rate difference in 25–34-year-olds, i.e. 1.23 per 100,000 population. Higher rates of HBsAg positivity among men have been described in several European countries [31-33]. Sex differences could be attributed to higher risk behaviour among men, particularly MSM. In the current study, male homosexual activity was reported as route of transmission in 25% of men. 

Sexual activity as the most important exposure and source of transmission is well recognised in other low-prevalence countries [22]. The observed low proportion of people who inject drugs (PWID) among the cases might be a result of decrease in heroin users in the country, needle and syringe exchange programmes or vaccination of PWID and their close contacts. Unfortunately, these data are not available in Belgium. During study follow-up, five mother-to-child infections were reported despite the recommendation of antenatal HBV screening in pregnant women, highlighting the need to strengthen their registration for HBV infection. A study conducted in Antwerp, Belgium indicated that HBsAg screening results were available at the moment of delivery for only 28% of the women who gave birth in 2010 [28]. It is crucial to be aware of the hepatitis B status of the mother as the child, in case of HBsAg positivity of the mother, will very likely be infected perinatally and with high risk of developing chronic hepatitis [13,34]. In line with the WHO recommendation, hepatitis B vaccination and immunoglobulins should be provided to the newborn within 24 hours if antenatal screening detects HBV infection, which in 94% is effective in preventing mother-to-child transmission [13,35].

Limitations of our study are intrinsic to the surveillance system in Flanders, i.e. the number of actual acute hepatitis B rates could be underestimated because of under-reporting and underdiagnosis. However, the acute hepatitis B notification rates from 2009 to 2017 in Flanders showed a downward trend. In the European Union, the average annual acute hepatitis B notification rate between 2006 and 2014 was 1.0 per 100,000 population [36]. The surveillance data for acute hepatitis B notification rates in Flanders were therefore comparable with other European countries, although direct comparison between countries is not possible considering the heterogeneity in acute hepatitis B case definitions and reporting systems. It should also be noted that the available surveillance data do not permit comparison of hepatitis B incidence before and after the introduction of universal hepatitis B vaccination. It is indeed not possible to distinguish the specific impact of hepatitis B vaccination from that of other prevention and control measures. Data on transmission routes were missing in 61% and in 17%, country of birth was unknown. The absence of these data could reduce statistical power and as missing data are random, we could consider randomness as not producing bias.

## Conclusion

Acute hepatitis B notification rates decreased in all age groups and declined from 1.56 per 100,000 population in 2009 to 0.66 in 2017. This may in part be explained by the universal vaccination in infants with catch-up in 10–13-year-olds, which can have a positive impact among adolescents and adults. The possible reversal of the decreasing trend for 15–24- and 25–34-year-olds in 2016 and 2015, respectively, could largely be explained by an increase in cases in MSM, demanding stronger prevention and control measures in adult risk-groups.
